# The validity and reliability of the Turkish version of the Patient’s Knee Implant Performance (PKIP) questionnaire-preoperative and postoperative

**DOI:** 10.1097/MD.0000000000042293

**Published:** 2025-04-25

**Authors:** Nilay Şahan, Songül Atasavun Uysal, Ayhan Parmaksiz, Erbil Aydin

**Affiliations:** a Department of Occupational Therapy, Faculty of Health Sciences, Çankiri Karatekin University, Çankiri, Turkey; b Faculty of Physical Therapy and Rehabilitation, Hacettepe University, Ankara, Turkey; c Department of Biostatistics, Faculty of Medicine, Istanbul Health and Technology University, Istanbul, Turkey; d Department of Orthopaedics and Traumatology, University of Health Sciences, Dişkapi Yildirim Beyazit Research and Training Hospital, Ankara, Turkey.

**Keywords:** functional performance, reliability and validity, total knee replacement

## Abstract

The Patient’s Knee Implant Performance (PKIP) Questionnaire is a short and easy-to-complete questionnaire developed to assess the performance of total knee arthroplasty (TKA) more comprehensively. The aim of this study was to investigate the validity and reliability of the Turkish version of the PIKP questionnaire before (PKIP PreOp) and after (PKIP PostOp) TKA. The study included 162 patients referred for TKA and 154 patients who had undergone the surgery at least 3 months prior. Cronbach alpha, intra-class correlation coefficient, and item-total correlation values were calculated to assess the reliability of the PIKP questionnaire. Validity was determined using exploratory and confirmatory factor analysis. To determine parallel scale validity, the *Western Ontario and McMaster Universities Osteoarthritis Index (WOMAC*), *The Short Form-36 (SF-36) Quality of Life Scale* performance score were used. The mean age of the participants was 66.39 ± 7.65 years. Cronbach alpha and intra-class correlation coefficient values of the PKIP were acceptable (0.723 and 0.985, respectively). The item-total correlation values of each item of the PKIP was also acceptable (lowest ranged from 0.335 to 0.621). Confirmatory and exploratory factor analysis revealed that the both PKIP PreOp and PKIP PostOp studies had sufficient fit. The PKIP PreOp and PKIP PostOp was moderately to strongly correlated with the Western Ontario and McMaster Universities Osteoarthritis Index and Short Form-36 score (*P* < .001). Patients undergoing TKA had a significantly higher PKIP PostOp score than PKIP PreOp score. The Turkish version of the PKIP is valid, reliable, and sensitive to assess in performance in patients undergoing TKA.

## 1. Introduction

Total knee arthroplasty (TKA) is a prevalent and efficacious treatment modality in orthopedic clinics, with the aim of alleviating pain and disability in the knee joint.^[1,2]^ In Turkey, there has been a notable increase in the annual number of reported TKA procedures.^[3]^ The increase in the number of TKA procedures can be interpreted as a positive sign of its effectiveness in safely reducing pain and improving functional status, especially in an aging population.^[4]^

In orthopedics, the objective assessment of outcomes after TKA is essential to determine the prognosis and to evaluate the effectiveness of the treatment.^[5]^ Although TKA is an efficacious procedure that improves patients’ quality of life,^[6]^ it is worth noting that 15% to 25% of patients report dissatisfaction, which has led to an increase in the use of questionnaires.^[1,7,8]^ Consequently, in order to assess the efficacy of TKA, it is vital to evaluate patient satisfaction and expectations with respect to activities of daily living in addition to the assessments conducted with clinical tools.^[9]^ Studies have also shown that patient satisfaction is associated with patients’ confidence and awareness, while knee function is influenced by balance, movement, satisfaction, and confidence in the knees of patients.^[10,11]^

Various questionnaires have been used to evaluate the results of TKA in studies in the literature, including the Knee injury and Osteoarthritis Outcome Score-Physical Function Short Form (KOOS-PS), Western Ontario and McMaster Universities Osteoarthritis Index (WOMAC), Forgotten Joint Score, 12-item Short Form Knee injury and Osteoarthritis Outcome Score (KOOS-12), the Oxford Knee Score, Lower Extremity Functional Scale, and Short Form 36 (SF-36), which measure patients’ functionality.^[12–15]^ The Patient’s Knee Implant Performance (PKIP) Questionnaire is a short and easy-to-complete questionnaire developed to assess the performance of TKA more comprehensively. Additionally, PIKP allows for the reportable evaluation of functionality in patients with TKA stability, mobility, satisfaction, and confidence.^[16]^

It is seen that there is a need for cultural adaptation, validity, and reliability of the PIKP Questionnaire that measures the performance of patients before and after TKA surgeries, which are also commonly performed in our country, due to the lack of a Turkish version of the scale. In this study, the objective was to translate the PIKP questionnaire into Turkish and assess its validity and reliability in a Turkish cultural context. The goal was to provide a scale that other researchers could utilize to evaluate their patients’ TKA performance processes.

## 2. Methods

The necessary ethics committee permission to conduct the research was obtained from the University of Health Sciences, with the decision number 136/15 dated April 25, 2022. The study was conducted May 2022 and April 2023 according to the principles of the Declaration of Helsinki.

### 2.1. Translation of the questionnaire

For the Turkish translation of the PKIP questionnaire, the methods and recommendations from Wild et al’s study were utilized. According to this methodology, two independent translators, who were both physiotherapists and academics, translated the questionnaire into Turkish, considering the cultural aspects of the target population. Later, a native English speaker translated the Turkish version into English, and a consensus was reached to obtain the final version. The comprehensibility of the final version of the questionnaire was evaluated by 10 individuals, and no difficulty in understanding any of the questions was encountered during the pilot test.^[17]^

### 2.2. Protocol

The study included 162 patients referred for TKA surgery by an orthopedist in Dişkapi Yildirim Beyazit Education and Research Hospital Orthopaedics and Traumatology Clinic and 154 patients who had undergone the operation at least 3 months prior. The study included individuals who were literate in Turkish and had a primary indication for TKA, with a diagnosis of primary osteoarthritis, and for the postoperative, had at least 3 months passed since TKA group. Patients who underwent TKA for reasons related to trauma, who had cognitive impairments, poor reading abilities, medical and psychiatric histories, postoperative complications that would affect their ability to function (such as infection), and those who did not consent to participate in the study were excluded.

Prior to surgery, detailed information about the purpose and method of the study was provided to 162 patients who met the inclusion criteria and agreed to participate, and informed consent forms were obtained from all patients participating in the study and their demographic information was recorded. The evaluation parameters of the study included the preoperative PKIP Questionnaire, the WOMAC, and the SF-36 Quality of Life Scale, which were administered at the Dişkapi Yildirim Beyazit Education and Research Hospital Orthopaedics and Traumatology Clinic. The assessments took approximately 30 to 40 minutes. Afterward, at the 3rd postoperative month, 154 patients were reached and asked to complete the postoperative PKIP questionnaire, the WOMAC, and the SF-36 Quality of Life Scale.

### 2.3. Assessment tools

*The PKIP Questionnaire-Preoperative and the PKIP Questionnaire-Postoperative:* PKIP are valid and reliable questionnaires consisting of 24 questions that allow the measurement of changes in knee function before and after TKA in a way that can be reported by the patient (*R* = 0.77). The questionnaire consists of 5 sections, which are knee awareness, confidence, stability, modify activities, and satisfaction. It also includes 5-point, 6-point, and 11-point rating scales. The questions in the questionnaire are categorized as follows: question 1 for knee awareness subheading, questions 3 and 4 for confidence subheading, questions 5 and 6 for stability subheading, question 7 for change in activities subheading, and questions 2, 8, and 9 for satisfaction subheading. Confidence, stability, and modify activities are evaluated with an 11-point numerical rating scale, while stability and frequency (joint awareness) are evaluated with a 5-point numerical rating scale, and satisfaction is evaluated with a 6-point numerical rating scale. The first question and the seventh question are reversed in the calculation of the total score obtained from the questionnaire. In the calculation of the questionnaire, each of the PKIP items is converted into a range of 0 to 10. The maximum score that can be obtained from the questionnaire is 100. Patients are asked to answer the questions based on their performance in the past week. High scores indicate that patients have high performance.^[11,16]^

*WOMAC:* WOMAC, the Turkish validity and reliability study of which was conducted by Tüzün et al,^[18]^ is a widely used and valid index for evaluating patients with osteoarthritis. The index comprises 24 questions and is organized into 3 sections, assessing pain, stiffness and physical function. Each question is scored with values from 0 to 4. 0 represents no symptoms, and 4 indicates severe symptoms. The total score for each section is determined separately, between 0 and 100. Higher scores indicate increased pain, stiffness and decreased physical function.^[18]^

*SF-36:* SF-36 was used to assess the quality of life of the patients. SF-36 scale includes 36 items and consists of 2 sections: physical and mental. The physical section consists of subscales of general health, physical function, role limitation due to physical function, and bodily pain, while the mental section consists of subscales of mental health, emotional role limitations, vitality/energy/fatigue, and social function. The subscales are scored from 0 to 100, with a score of “0” indicating poor health. The scale also includes items that question the patient’s perception of changes in their health over the past 4 weeks and the last week. Turkish validity and reliability of scale was conducted by Koçyiğit et al.^[19]^

### 2.4. Statistical analysis

In the literature, it is recommended to conduct studies at least 5 times the number of items, and if possible, 10, 15, or 20 times the number of items. In our study, it was aimed to collect data at least 5 times and 162 patients were reached. The study was conducted with approximately 7 times the number of items (162/24 = 6.75). In addition, Holter n number calculated for sample size adequacy in the confirmatory factor analysis phase is given below the relevant tables.^[20,21]^ Construct validity was assessed using the confirmatory factor analysis, while discriminant validity was evaluated through item analysis and item discrimination index calculations for the lower-upper 27% group. Additionally, the Cronbach Alpha coefficient was calculated to measure internal consistency and reliability. Values of 0.7 for Cronbach alpha, 0.20 for item-total correlation are considered as minimum acceptable values.^[22,23]^ Standardized coefficient estimates and model fit indices were obtained as a result of the confirmatory factor analysis. The estimation method was unweighted least squares. Model fit indices for confirmatory factor analysis included χ2/df (degrees of freedom), root mean square error of approximation (RMSEA), comparative fit index (CFI), normed fit index (NFI), standardized square root mean residual (SRMR), non-normed fit index (NNFI), and goodness of fit index (GFI). The model fit was accepted as good these values were accepted as acceptable if the value of the χ2/df was under 2 and higher than 0.90 for fit indices.^[24]^ To assess convergent validity, Pearson correlation coefficients were computed between PKIP and related scales (WOMAC and SF-36). The discrimination index was assessed by comparing the 27% lower and upper groups in the PKIP score using an independent samples *t*-test. Additionally, the study examined the floor-ceiling effect. A ceiling or floor effect was accepted as 15% (or more) of individuals in a sample achieving the best or the worst level of the score.^[25]^ Although there are no clear ranges for interpreting the magnitude of correlation coefficients, they can be interpreted based on their absolute value. If 0.00 ≤ *R* ≤ 0.25 the correlation is considered very weak; if 0.26 ≤ *R* ≤ 0.49 the correlation is considered weak; if 0.50 ≤ *R* ≤ 0.69 the correlation is considered moderate; 0.70 ≤ *R* ≤ 0.89 indicates a high correlation, and 0.90 ≤ *R* ≤ 1.00 indicates a very high correlation between the variables.^[26]^ A *P*-value of <.05 was considered significant. JASP (JASP Team, University of Amsterdam, Amsterdam, Netherlands, Version 0.16.1) was used for statistical analysis.

## 3. Results

**Figure 1.**
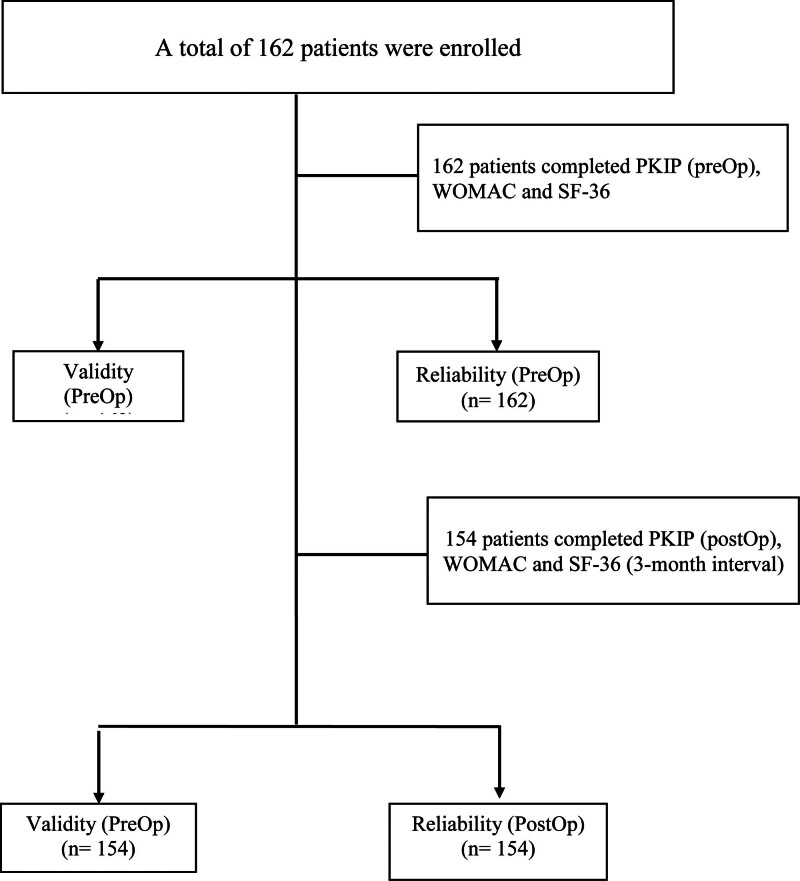
Flow diagram of the study participants.

One hundred sixty-two participants were included in the study and 116 (71.61%) were female. The participants had a mean age of 66.39 ± 7.65 years (min: 37–max: 80 years) (Table 1; Fig. 1).

**Table 1:** Patient characteristics.

	Number	Percentage
Sex		
Female	116	71.61
Male	46	28.40
The operated extremity		
Right	75	46.30
Left	86	53.09
Education		
Literate	23	14.20
Primary Education	129	79.63
High School	6	3.70
Üniversity	4	2.47
Age, mean ± SD (Min–Max)	66.39 ± 7.65 (37–80)	
BMI, mean ± SD (Min–Max)	31.65 ± 4.78 (19.85–46.87)	

BMI = body mass index.

**Figure 2.**
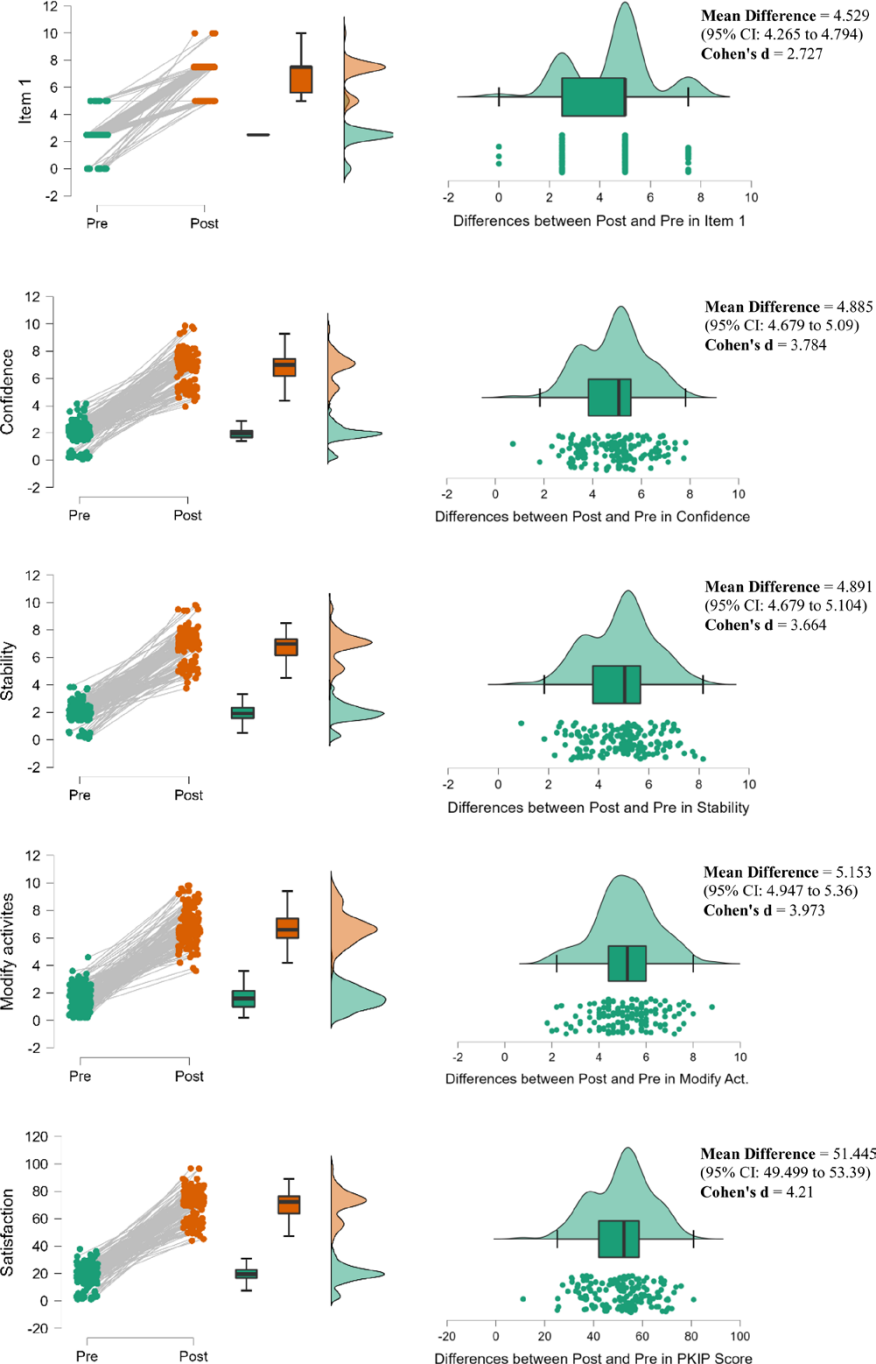
Distributions of PreOp, PostOp and difference between PostOp and PreOp Scores in PKIP.

To examine the ceiling and floor effects, we analyzed the PreOp and PostOp overall scores. Our results indicated that there was no accumulation of scores at the lowest and highest ends, as shown in the histogram graphs of PreOp and PostOp studies in the last row of Figure 2.

### 3.1. Reliability analyses

Tables 2 and 3 show Cronbach alpha statistics for the different factors in the PreOp and PostOp studies. The Cronbach alpha statistics in the PreOp and PostOp studies were 0.927 and 0.965 for the confidence factor, 0.908 and 0.972 for the stability factor, 0.896 and 0.950 for the modify activities factor, 0.723 and 0.871 for the satisfaction factor, and 0.958 and 0.985 for the overall score, respectively (Tables 2 and 3).

**Table 2:** Item analysis, confirmatory factor analysis and discriminant index in PKIP-PreOp (n = 162).

Factor	Item	Item analysis					CFA*		Discrimination index	
		Mean	SD	Item-rest correlation	If item dropped CA	CA	Std. Est.	*R*²		Mean difference† (95% CI)
Knee awareness	Item1	2.423	1.058	–	–	–	–	–		1.648 (2.148–1.148)
Confidence		1.609	0.731			0.927				
	Item3a	1.858	1.074	0.783	0.915		0.834	0.695		1.886 (2.241–1.532)
	Item3b	0.846	0.808	0.832	0.912		0.827	0.684		1.432 (1.697–1.167)
	Item3c	1.019	0.830	0.814	0.913		0.839	0.705		1.5 (1.785–1.215)
	Item3d	2.117	0.887	0.755	0.917		0.803	0.645		1.636 (1.956–1.317)
	Item3e	1.802	0.855	0.804	0.913		0.835	0.698		1.568 (1.843–1.293)
	Item3f	1.074	0.846	0.831	0.911		0.846	0.716		1.591 (1.878–1.304)
	Item3g	1.796	0.893	0.819	0.912		0.839	0.704		1.591 (1.899–1.283)
	Item4	2.361	0.975	0.442	0.942		0.513	0.264		1.42 (1.892–0.949)
Stability		1.563	0.730			0.908				
	Item5a	2.000	1.015	0.811	0.885		0.859	0.738		1.841 (2.166–1.515)
	Item5b	1.778	0.972	0.841	0.881		0.878	0.771		1.841 (2.152–1.53)
	Item5c	0.969	0.874	0.865	0.880		0.874	0.764		1.568 (1.855–1.282)
	Item5d	0.877	0.862	0.860	0.880		0.855	0.731		1.545 (1.811–1.28)
	Item5e	0.784	0.794	0.812	0.887		0.827	0.685		1.386 (1.637–1.136)
	Item5f	2.130	0.820	0.648	0.903		0.712	0.506		1.25 (1.529–0.971)
	Item6	2.407	1.000	0.335	0.939		0.476	0.227		1.477 (1.961–0.994)
Modify activities		1.620	0.778			0.896				
	Item7a	1.599	0.859	0.662	0.890		0.671	0.45		1.273 (1.589–0.956)
	Item7b	2.235	0.975	0.736	0.875		0.745	0.556		1.568 (1.913–1.223)
	Item7c	1.420	0.983	0.763	0.869		0.899	0.809		1.818 (2.151–1.485)
	Item7d	1.093	0.862	0.781	0.865		0.868	0.754		1.545 (1.839–1.252)
	Item7e	1.753	0.946	0.781	0.864		0.783	0.613		1.455 (1.82–1.09)
Satisfaction		1.638	0.804			0.723				
	Item2	1.123	0.995	0.629	0.530		0.73	0.533		1.864 (2.019–1.708)
	Item8	2.531	1.042	0.410	0.797		0.637	0.405		1.477 (1.966–0.989)
	Item9	1.259	0.969	0.611	0.556		0.689	0.475		1.773 (1.971–1.574)
Overall		1.636	0.663			0.958				

CA = Cronbach Alpha, CFA = confirmatory factor analysis, CI = confidence interval, PKIP = Patient’s Knee Implant Performance Questionnaire, SD = standard deviation, Std. Est = standardized estimates.

* Estimation Method: Unweighted Least Squares (ULS); Goodness of Fit Indices: χ2/df = 238.59/224 = 1.065; RMSEA = 0.020; SRMR = 0.081; NFI = 0.970; NNFI = 0.998; CFI = 0.998; GFI = 0.978; Hoelter critical N (α = .05) = 176.39.

† Mean difference between 27% upper and lower groups in PKIP PreOp Score.

**Table 3:** Item analysis, confirmatory factor analysis and discriminant index in PKIP-PostOp (n = 154).

Factor	Item	Item analysis					CFA*		Discrimination index
		Mean	SD	Item-rest correlation	If item dropped CA	CA	Std. Est.	*R*²	Mean difference† (95% CI)
Knee awareness	Item2.1	6.932	1.196	-	-	–	–	–	2.44 (2.78–2.101)
Confidence		6.711	1.187			0.965			
	Item2.3a	7.266	1.161	0.912	0.959		0.922	0.85	2.548 (2.867–2.228)
	Item2.3b	5.188	1.656	0.775	0.969		0.813	0.66	3.024 (3.542–2.506)
	Item2.3c	6.494	1.354	0.939	0.956		0.942	0.887	2.905 (3.322–2.488)
	Item2.3d	6.942	1.397	0.918	0.957		0.927	0.858	3.048 (3.451–2.644)
	Item2.3e	7.240	1.205	0.925	0.958		0.924	0.854	2.571 (2.947–2.196)
	Item2.3f	6.416	1.307	0.920	0.957		0.937	0.879	2.881 (3.254–2.508)
	Item2.3g	7.175	1.227	0.920	0.958		0.917	0.84	2.571 (2.962–2.181)
	Item2.4	6.964	1.211	0.681	0.970		0.744	0.553	2.56 (2.899–2.22)
Stability		6.686	1.190			0.972			
	Item2.5a	7.247	1.145	0.933	0.965		0.941	0.886	2.524 (2.864–2.184)
	Item2.5b	6.981	1.291	0.945	0.964		0.953	0.908	2.833 (3.218–2.449)
	Item2.5c	6.468	1.289	0.943	0.964		0.946	0.894	2.738 (3.155–2.321)
	Item2.5d	6.260	1.303	0.924	0.965		0.921	0.849	2.619 (3.074–2.164)
	Item2.5e	5.987	1.304	0.932	0.965		0.948	0.898	2.786 (3.197–2.375)
	Item2.5f	6.877	1.425	0.901	0.967		0.929	0.863	3.119 (3.532–2.706)
	Item2.6	6.981	1.235	0.715	0.979		0.759	0.577	2.619 (2.974–2.264)
Modify activities		6.774	1.207			0.950			
	Item2.7a	5.643	1.579	0.815	0.953		0.85	0.723	2.952 (3.475–2.43)
	Item2.7b	7.175	1.211	0.841	0.943		0.866	0.75	2.286 (2.737–1.835)
	Item2.7c	7.169	1.225	0.912	0.931		0.924	0.853	2.548 (2.929–2.166)
	Item2.7d	6.831	1.287	0.887	0.934		0.914	0.836	2.548 (2.983–2.112)
	Item2.7e	7.052	1.272	0.899	0.932		0.931	0.867	2.714 (3.104–2.325)
Satisfaction		7.900	1.135			0.871			
	Item2.2	8.416	1.307	0.830	0.744		0.84	0.706	2.429 (2.872–1.985)
	Item2.8	6.831	1.226	0.621	0.929		0.831	0.691	2.512 (2.812–2.212)
	Item2.9	8.455	1.284	0.820	0.754		0.832	0.692	2.429 (2.856–2.002)
Overall		6.874	1.118			0.985			

CA = Cronbach Alpha, CFA = confirmatory factor analysis, CI = confidence interval, PKIP = Patient’s Knee Implant Performance Questionnaire, SD = standard deviation, Std. Est = standardized estimates.

* Estimation Method: Unweighted Least Squares (ULS); Goodness of Fit Indices: χ2/df = 276.36/224 = 1.234; RMSEA = 0.039; SRMR = 0.048; NFI = 0.996; NNFI = 0.999; CFI = 0.999; GFI = 0.996; Hoelter critical N (α = .05) = 144.897.

† Mean difference between 27% upper and lower groups in PKIP PostOp Score.

We also evaluated item-rest correlation in the item analysis. The lowest item-rest correlation was 0.335 in the PreOp study, while it was 0.621 in the PostOp study, as shown in Tables 2 and 3.

### 3.2. Validity analysis

We performed validity analyses using construct validity and convergent-divergent validity analyses.

#### 3.2.1. Construct validity

The standardized estimates obtained in confirmatory factor analysis were statistically significant at the 0.001 significance level for both PreOp and PostOp studies. The lowest R-square was 0.227 in the PreOp study and 0.553 in the PostOp study. The results of the confirmatory factor analysis indicated a good fit. The GFIs were χ2/df = 1.065, RMSEA = 0.020, SRMR = 0.081, NFI = 0.970, NNFI = 0.998, CFI = 0.998, and GFI = 0.978 in the PreOp study, and χ2/df = 1.234, RMSEA = 0.039, SRMR = 0.048, NFI = 0.996, NNFI = 0.999, CFI = 0.999, and GFI = 0.996 in the PostOp study (Tables 2 and 3).

Additionally, the discrimination indices for all items were found to be statistically significant in both PreOp and PostOp studies (Tables 2 and 3).

#### 3.2.2. Convergent-divergent validity

Given the correlation data in Table 4, there was a strong negative correlation between the total score of PKIP Questionnaire before total knee arthroplasty and the pretreatment total score of WOMAC (*R* = −0.7; *P* < .001).

**Table 4:** Relationship between PKIP-Preop. and scale scores.

	Mean ± SD	Knee awareness 2.42 ± 1.06	Confidence 1.93 ± 0.74	Stability 1.92 ± 0.73	Modify activities 1.62 ± 0.78	Satisfaction 1.64 ± 0.8	PKIP-PreOp total 19.05 ± 6.89
Womac/pain	15.4 ± 2.42	−0.455***	−0.58***	−0.544***	−0.383***	−0.572***	−0.6***
Womac/stiffness	5.93 ± 1.21	−0.174*	−0.317***	−0.296***	−0.255**	−0.267***	−0.304***
Womac/physical function	51.08 ± 7.03	−0.531***	−0.626***	−0.617***	−0.454***	−0.606***	−0.672***
Womac/total	75.11 ± 9.9	−0.534***	−0.661***	−0.645***	−0.494***	−0.623***	−0.7***
SF-36/ physical functioning	19.35 ± 7.71	0.546***	0.632***	0.623***	0.434***	0.564***	0.665***
SF-36/ role physical	17.44 ± 21.96	0.526***	0.57***	0.562***	0.394***	0.506***	0.611***
SF-36/ role emotional	35.78 ± 29.38	0.422***	0.4***	0.395***	0.211**	0.33***	0.424***
SF-36/ vitality	31.2 ± 10.01	0.463***	0.569***	0.573***	0.583***	0.549***	0.646***
SF-36/ mental health	47.62 ± 7.3	0.543***	0.563***	0.582***	0.425***	0.477***	0.618***
SF-36/ social functioning	30.25 ± 12.12	0.501***	0.647***	0.65***	0.428***	0.549***	0.656***
SF-36/ bodily pain	21 ± 11.59	0.608***	0.725***	0.719***	0.492***	0.702***	0.77***
SF-36/ general health	47.22 ± 12.81	0.563***	0.639***	0.639***	0.375***	0.592***	0.669***

PKIP = Patient’s Knee Implant Performance Questionnaire, SF-36 = The Short Form 36 (SF-36) Quality of Life Scale, WOMAC = Western Ontario and McMaster Universities Osteoarthritis Index.

* *P* < .05.

** *P* < .01.

*** *P* < .001.

On the other hand, the analysis of the correlation between the total score of PKIP Questionnaire before total knee arthroplasty and SF-36 in the pretreatment evaluations, which mainly evaluate the patient’s functionality, revealed a moderate to strong positive correlation with the physical functioning, social functioning, bodily pain, and general health subscales (*R* = 0.656–0.77; *P* < .001).

The correlation analyses after total knee replacement surgery showed a significant negative correlation between the total score of PKIP Questionnaire after total knee arthroplasty and the posttreatment total score of WOMAC (*R* = −0.872; *P* < .001) (Table 5).

**Table 5:** Relationship between PKIP-Postop. and scale scores.

	Mean ± SD	Knee awareness2 6.93 ± 1.2	Confidence2 6.82 ± 1.12	Stability2 6.81 ± 1.14	Modify activities2 6.77 ± 1.21	Satisfaction2 7.9 ± 1.14	PKIP-postop total 70.47 ± 10.58
Womac/pain2	4.07 ± 2.47	−0.678***	−0.737***	−0.722***	−0.595***	−0.785***	−0.769***
Womac/stiffness2	1.84 ± 1.21	−0.517***	−0.572***	−0.57***	−0.504***	−0.566***	−0.598***
Womac/physical function2	14.66 ± 8.44	−0.712***	−0.857***	−0.833***	−0.751***	−0.78***	−0.861***
Womac/total2	21.47 ± 11.96	−0.733***	−0.857***	−0.835***	−0.739***	−0.819***	−0.872***
SF-36/physical functioning2	76.53 ± 12.94	0.727***	0.833***	0.825***	0.735***	0.776***	0.853***
SF-36/ role physical2	88.31 ± 18.34	0.663***	0.661***	0.663***	0.534***	0.586***	0.681***
SF-36/ role emotional2	95.67 ± 11.26	0.504***	0.487***	0.496***	0.387***	0.532***	0.527***
SF-36/ vitality2	69.22 ± 11.39	0.645***	0.793***	0.792***	0.795***	0.67***	0.81***
SF-36/ mental health2	78.44 ± 8.73	0.472***	0.544***	0.539***	0.529***	0.508***	0.568***
SF-36/ social functioning2	81.04 ± 17.31	0.725***	0.771***	0.772***	0.637***	0.717***	0.793***
SF-36/ bodily pain2	74.72 ± 15.9	0.741***	0.799***	0.794***	0.662***	0.768***	0.824***
SF-36/ general health2	75.84 ± 10.55	0.614***	0.712***	0.711***	0.666***	0.633***	0.731***

PKIP = Patient’s Knee Implant Performance Questionnaire, SF-36 = The Short Form 36 (SF-36) Quality of Life Scale, WOMAC = Western Ontario and McMaster Universities Osteoarthritis Index.

*P < .05.

**P < .01.

*** *P* < .001.

Additionally, a strong positive correlation was found between the total score of PKIP Questionnaire after total knee arthroplasty and the posttreatment subscales of the SF-36, including physical functioning, social functioning, bodily pain, and general health (*R* = 0.731–0.853; *P* < .001) (Table 5).

## 4. Discussion

This study investigating the Turkish validity and reliability of the PKIP questionnaire before and after TKA surgery demonstrated that all sections of the questionnaire were easily understandable.

With the increasing number of TKA surgeries performed each year, questionnaires are used to assess the activity difficulty level and symptom severity of patients to understand their postoperative functionality. However, certain factors that can affect a patient’s performance, such as their perceived knee stability and balance, cannot be fully measured.^[8]^ As a result, the PKIP Questionnaire was developed as a valid and reliable tool to assess patient performance before and after TKA surgeries, taking into account factors such as instability and insecurity that may impact patient performance.^[11]^

Based on previous studies, it has been reported that elderly and postmenopausal women account for 70% to 90% of TKA candidates.^[27–29]^ This is attributed to estrogen deficiency in postmenopausal women, which predisposes to osteoporosis and osteoarthritis with poor bone quality and consequent dysfunction of the knee joint.^[30,31]^ Studies have shown that women with osteoarthritis have more pain sensitivity and severity and a higher psychosocial perception of disability than men, which leads to a lower quality of life.^[30,32]^ Gustayson et al analyzed the gender differences in recovery after TKA and performed repeated measurements at 1, 3, and 6 months. The study revealed that, at 1 month after TKA, women exhibited superior muscle strength retention in comparison to men; however, they demonstrated a more pronounced decline in physical functionality. However, these observed differences between the sexes did not persist at 3. and 6. months post-surgery.^[30]^ In accordance with the extant literature, the present study’s cohort comprised predominantly female patients who underwent TKA surgery, exhibiting a significant improvement in our measurements at the 3-month mark.

The researchers who developed the PKIP Questionnaire stated that the reliability of the questionnaire ranged between 0.70 and 0.90, with the reliability of the subscales being above 0.90, excluding the stability and modify activity subscales. In this study, the reliability of the PKIP Questionnaire was assessed using Cronbach alpha values. The results showed that the alpha values were above 0.90 for the subscales of confidence, stability, and modify activities, excluding the satisfaction subscale. The Cronbach alpha value for the satisfaction subscale was 0.723. These results indicate that the reliability of the PKIP Questionnaire in this study is consistent with the results reported by the authors who developed the questionnaire and that all the subscales of the questionnaire are reliable.

In the item analysis evaluation of the questionnaire, although it was observed that the alpha value increased with the removal of some items, these items did not weaken the psychometric properties of the questionnaire (they were within the required limits) and no items were removed by preserving the natural structure of the questionnaire with the idea that the questionnaire questioned the evaluation of the patients in the direction of recovery in more detail.

In addition, since the scale questions were administered to the patient group preoperatively and at 3 months postoperatively and the patients received physiotherapy treatment postoperatively, a suitable time interval for test-retest measurement was not possible.

The authors of the PKIP Questionnaire stated in their study that no ceiling or floor effects or other response biases were observed in the preoperative and postoperative patient evaluations (16). Similarly, in this study, no ceiling or floor effect was observed in the lowest and highest scores of the preoperative and postoperative general scores of the PKIP Questionnaire, in line with the results of the researchers who developed the questionnaire.

The researchers who created the questionnaire only reported the results of the confirmatory factor analysis for postoperative evaluations in their study and suggested that the five subscale scores should be combined to form the overall score of the PKIP Questionnaire. However, in this study, we performed a confirmatory factor analysis for both preoperative and postoperative evaluations and found that the standardized estimates were statistically significant at the 0.001 significance level. The lowest R-square value was 0.227 in the PreOp study and 0.553 in the PostOp study. All the fit indices obtained from the confirmatory factor analysis were at least acceptable, and the collected data demonstrated that the construct validity of the model was established.

In this study, evaluations were performed before and 3 months after surgery. The results revealed a statistically significant improvement in patients’ conditions. Furthermore, the effect size of the difference between preoperative and postoperative evaluations was high, which is consistent with the findings of the researchers who developed the questionnaire. This is considered to be satisfactory in terms of observing the change before and after treatment. However, it was noted that most patients were unable to perform a squat activity before the operation and reported fear of performing it after the operation. This is valuable in terms of the purpose and result of the study. Because squatting is an essential position for prayer in our culture, many patients still reported difficulty squatting. This underscores the importance of educating patients about post-surgery activities they can perform. Additionally, based on the researchers’ recommendations, it is advisable to evaluate patients 1 year after surgery.

In their study, the authors who developed the PKIP Questionnaire correlated the pre and postoperative results with other questionnaires (KOOS, Oxford knee score, American Knee Society, three-level EuroQol five-dimensional questionnaire, Clinical Global Impression) that assessed patients’ symptoms and quality of life. They only used the total scores of the measurements and obtained moderate-to-high correlation results. In this study, we compared the preoperative and postoperative evaluations of the PKIP with the preoperative and postoperative evaluations of WOMAC and SF-36. We found that the postoperative evaluations of the patients in all measurements yielded much better results in terms of improvement than the preoperative evaluations. When we evaluated the preoperative and postoperative results of the PKIP Questionnaire, WOMAC, and SF-36 in terms of their correlation with all subscales, such as physical functioning, social functioning, bodily pain, and general health, which are especially related to functionality, we found a moderate-to-strong correlation between the results of the subscales. These results indicate that the PKIP Questionnaire is consistent in questioning the functionality of patients before and after TKA.

In their study on questionnaires reported by patients undergoing TKA, Wang et al^[33]^ evaluated the questionnaires according to the COSMIN^[15,34,35]^ method, which is based on factors such as validity, reliability, degree of bias, and the standards of the questions to be directed to the patient. As a result of their studies, they stated that the PKIP questionnaire consisted of specific and comprehensive approaches to evaluate the content validity and that it has a sufficient degree of methodological quality. We believe that the preoperative and postoperative evaluations of the PKIP questionnaire are comprehensive in terms of questioning the satisfaction and functionality of Turkish patients.

In addition, when the literature is examined, it is seen that the Japanese language validity and cultural adaptation of the PKIP Questionnaire have been made and used as an evaluation criterion in the studies.^[36,37]^

However, this study has some limitations. All questionnaires (PKIP, WOMAC, and SF-36) were administered in the same evaluation session for both preoperative and postoperative cases, which may have caused a respondent burden that could have affected the responses of the patients. Additionally, the majority of the study sample consisted of elderly female patients, which may have resulted in differences in pain thresholds, activity levels, and satisfaction levels between genders. Therefore, further studies comparing the results with male patients or the results by gender may be necessary.

In conclusion, the study demonstrated that the Turkish version of the PKIP questionnaire is a valid and reliable tool for assessing the preoperative and postoperative performance and satisfaction of Turkish-speaking patients who undergo TKA.

## Acknowledgments

The authors would like to thank the subjects who voluntarily participated in the study.

## Author contributions

**Conceptualization:** Nilay Şahan, Songül Atasavun Uysal.

**Data curation:** Nilay Şahan.

**Formal analysis:** Ayhan Parmaksiz.

**Investigation:** Nilay Şahan.

**Methodology:** Nilay Şahan, Songül Atasavun Uysal.

**Resources:** Nilay Şahan, Erbil Aydin.

**Supervision:** Songül Atasavun Uysal, Erbil Aydin.

**Writing – original draft:** Nilay Şahan, Songül Atasavun Uysal.

**Writing – review & editing:** Nilay Şahan, Erbil Aydin.
